# Serum TSH levels as a predictor of malignancy in thyroid nodules: A prospective study

**DOI:** 10.1371/journal.pone.0188123

**Published:** 2017-11-16

**Authors:** Lenara Golbert, Ana Patrícia de Cristo, Carlo Sasso Faccin, Mauricio Farenzena, Heloísa Folgierini, Marcia Silveira Graudenz, Ana Luiza Maia

**Affiliations:** 1 Endocrine Division, Santa Casa de Porto Alegre, Universidade Federal de Ciências da Saúde de Porto Alegre, Porto Alegre, RS, Brasil; 2 Thyroid Section, Endocrine Division, Hospital de Clínicas de Porto Alegre, Universidade Federal do Rio Grande do Sul, Porto Alegre, RS, Brasil; 3 Radiology Division, Hospital de Clínicas de Porto Alegre, Universidade Federal do Rio Grande do Sul, Porto Alegre, RS, Brasil; 4 Pathology Division, Hospital de Clínicas de Porto Alegre, Universidade Federal do Rio Grande do Sul, Porto Alegre, RS, Brasil; 2nd medical school of Charles University, CZECH REPUBLIC

## Abstract

**Background:**

The role of serum TSH concentrations as a predictor of malignancy of thyroid nodule remains unclear.

**Objective:**

To prospectively evaluate the usefulness of serum TSH levels as a predictor of malignancy in thyroid nodules.

**Methods:**

Patients with thyroid nodule(s) who underwent fine-needle aspiration biopsy under ultrasonographic guidance in a tertiary, university-based hospital were consecutively evaluated. Patients with known thyroid cancer and/or patients receiving thyroid medication were excluded. Serum TSH levels were measured by two differents methodologies, chemiluminescent (CLIA) and electrochemiluminscent immunoassay (ECLIA). Anatomopathological exam of tissue samples obtained at thyroidectomy was considered the gold standard for the diagnosis of thyroid cancer.

**Results:**

A total of 615 patients participated in the study. The mean age was 55.9±14.7 years, and 544(88.5%) were female. The median TSH values were 1.48 and 1.55 μU/mL, using CLIA and ECLIA, respectively. One-hundred-sixty patients underwent thyroidectomy and the final diagnoses were malignant in 47(29.4%) patients. TSH levels were higher in patients with malignant than in those with benign nodules in both TSH assays: 2.25 vs. 1.50; P = 0.04 (CLIA) and 2.33 vs. 1.27; P = 0.03 (ECLIA). Further analysis using binary logistic regression identified elevated TSH levels, a family history of thyroid cancer, the presence of microcalcifications, and solitary nodule on US as independent risk factors for malignancy in patients with thyroid nodules. Additional analyses using TSH levels as a categorical variable, defined by ROC curve analysis, showed that the risk of malignancy was approximately 3-fold higher in patients with TSH levels ≥2.26 μU/mL than in patients with lower TSH levels (P = 0.00).

**Conclusions:**

Higher serum TSH levels are associated with an increased risk of thyroid cancer in patients with thyroid nodules. Using TSH levels as an adjunctive diagnostic test for stratifying the risk of malignancy associated with a thyroid nodule may help on defining the best therapeutic approaches.

## Introduction

Palpable thyroid nodules are a common disorder detected in 4–7% of adults in the general population and in 19–67% of patients who undergo high-resolution ultrasound [1–5]. In contrast, thyroid cancer is rare. However, in most cases, thyroid carcinoma presents clinically as a nodule (solitary or in a multinodular gland) that is indistinguishable from benign neoplasia. The challenge for clinicians, therefore, is to distinguish malignant (5–10%) from benign thyroid nodules[4,5]. Of note, the last National Cancer Institute State Cancer Profile has shown that the incidence of thyroid cancer is rising faster than that of any other malignant neoplasia[6].

Fine-needle aspiration biopsy (FNAB) is the gold standard for evaluating patients with thyroid nodules, and it is currently the most reliable diagnostic technique for evaluating thyroid nodules under ultrasound guidance[5,7]. Diagnostic results are obtained in most cases, as inadequate specimens (nondiagnostic or unsatisfactory) occur in only 5–10% of cases. However, FNAB cannot reliably rule out cancer in 20–30% of nodules, reported as indeterminate for malignancy [8–10] The Bethesda classification [11] recognizes three specific cytological diagnoses characterized by indeterminate cytology. The predicted probability of cancer is 5–10% in Bethesda III patients, 20–30% in Bethesda IV patients, and 50–75% in Bethesda V patients, but variability has been noted at different centers [8,10] and most patients with indeterminate cytology undergo surgery to establish the histopathologic diagnosis. However, only 10–20% of the thyroid nodules with indeterminate cytology (Bethesda III and IV) are malignant[8,12–14]. New diagnostic approaches based on thyroid cancer molecular biomarkers have recently been studied, and some are already introduced in clinical settings. Currently, the most successful panels testing for mutations in thyroid FNAB samples are those testing for BRAF and RAS point mutations and RET/PTC and PAX8/PPARγ rearrangements, as well as TRK rearrangements [15, 16]. Although the use of these molecular tools have been validated in some studies, these tests are expensive (and not cost-effectivity depending of thyroid cancer prevalence), and their impact on patient management remains debatable[17–20].

The role of TSH as a predictor of thyroid nodule malignancy has been evaluated by several studies in the last years. Since Boelaert has reported parallel increases in malignancy risk and serum TSH levels[21], several other authors have investigated the relationship between serum TSH levels and thyroid cancer with conflicting results[22–24]. A recent meta-analysis found a positive association between higher serum TSH levels and increased risk of thyroid cancer[25]. However, the analysis had some limitations, as all of its included studies were cross-sectional, and the vast majority was retrospective (only two prospective studies). In contrast, the EPIC study, which is based on a huge European cohort, demonstrated a negative association between TSH levels and thyroid cancer risk[26]. Although the EPIC study is a large prospective study, it is a case-control study featuring control subjects who were chosen from a group of cancer-free participants constituting the EPIC cohort (healthy population), thus, which may limit its conclusions.

Here, we aimed to evaluate the role of serum TSH levels as predictor of thyroid nodule malignancy in a cohort of patients with thyroid nodules in a tertiary, university-based hospital.

## Materials and methods

Consecutive patients with thyroid nodule(s) who underwent FNAB under ultrasonographic guidance (US-FNAB) between 2012 and March of 2016 in the Thyroid Unit of the Hospital de Clínicas de Porto Alegre, a tertiary, university-based hospital, were prospectively evaluated. The geographical area of the study is iodine sufficient[27]. All patients were referred for evaluation of thyroid nodules and underwent a complete history and physical examination. Patients with known thyroid cancer and/or patients receiving thyroid-related medication were excluded. TSH levels, ultrasound characteristics of the nodules, demographic and clinical data were compared in patients with benign and malignant thyroid nodule. The project was approved by the Research Ethics Committee of the Hospital de Clínicas de Porto Alegre (GPPG 140538). The study was conducted in accordance with the ethical standards of the local institutional, national research committee and with the Helsinki declaration. The local institutional and national research committee did not request informed consent, as the data were analyzed anonymously and the assistance of patients follow routine clinical indications.

### Laboratory evaluation

Serum TSH levels were measured by two different methodologies, chemiluminescent immunoassay (CLIA) (ADVIA Centaur XP; Siemens, Tarrytown, NY), with interassay coefficient of variation of 5.3% over the range 0.35–5.50 μU/mL, were used until November 2014. After that, TSH levels were measured by electrochemiluminscent immunoassay (ECLIA) (Roche Diagnóstica, São Paulo, Brasil), with interassay coefficient of variation of 3.11%, reference values: 0.27–4.20 uUI/mL. The thyroid peroxidase antibodies (TPO-Ab) was evaluated by chemiluminescent immunoassay (IMMULITE Systems, Siemens Healthcare Diagnostics, Tarrytown, NY), reference value inferior of 34.0UI/mL.

### Ultrasound examination

All patients underwent thyroid US-FNAB, which was performed by the same radiologist using a high-resolution ALOKA ultrasound device with a 7.5 MHz linear transducer (Tokyo, Japan). The number of thyroid nodules, as well as sizes, US characteristics (echogenicity, the presence of microcalcification, halos, contours, and shape), and the presence of cervical lymphadenopathy were documented in the medical records of all patients.

### FNAB, cytological and histological diagnosis

*FNA and cyto-cell block testing*. US-FNA was performed with a disposable needle (21G) connected to a 10 ml disposable syringe[14]. Multidirectional aspiration was performed in dominant nodules in patients with multinodular goiters and/or suspicious nodules on ultrasonography[5,7]. Rapid on-site evaluations of all fine-needle aspiration specimens were performed to determine the adequacy of each specimen. A thyroid FNA specimen was considered satisfactory if at least 6 groups of follicular cells were present, and each group comprised at least 10 cells. Immediate on-site reaspiration was performed in cases in which specimens considered inadequate for diagnosis were obtained. Six cytology slides were prepared for each patient, four of which were air-dried and immediately stained via the May Grümwald Giemsa technique. The other two slides were immediately fixed in ethanol 96° and subsequently stained via the Papanicolaou technique. The residual hemorrhagic aspirated in the syringe and needle was fixed in 10% formalin. Then, the aspirated material was centrifuged, paraffin-embedded (cell block), sectioned and stained with hematoxylin and eosin to serve as a complementary diagnostic specimen to the FNA specimen. A sample was considered viable if it contained a sufficient number of cells with intact morphology (60 cells from at least 6 groups of 10 follicular cells).

Two independent pathologists reviewed the cytological and histological slides of each case together and assigned the samples to a final diagnostic category. Cytological aspirate adequacy was defined according to the recommendations of the *Papanicolaou Society of Cytopathology Task Force on Standards of Practice* (1996), and the cytological results were classified into the following 6 diagnostic categories according to the criteria of the Bethesda System for Cytological Classification of Thyroid Nodules: 1) non-diagnostic or unsatisfactory, 2) benign, 3) atypia of undetermined significance, 4) a follicular neoplasm or suspicious for a follicular neoplasm, 5) suspicious for malignancy and 6) malignant.

Cell-block slides were reviewed for the presence of cellular elements and classified into the following two categories: 1) diagnostic and 2) non-diagnostic. Cell-block US-FNBA specimens were used as adjunctive diagnostic specimens, and the results were described as cyto-cell blocks[14].

Anatomopathological examinations of tissue samples obtained at thyroidectomy were carried out according to the World Health Organization Guidelines, and the pathology reports pertaining to these samples were considered the gold standard for the diagnosis of thyroid cancer. The pathology reports provided information regarding AJCC/UICC TNM staging and the presence of invasion. The risk of recurrence was evaluated according to the ATA thyroid cancer guidelines[5,7].

### Statistical analysis

The clinical, laboratory, ultrasonography and cytological data, which are reported as the mean–standard deviation (SD) values, or as the median with percentiles 25 and 75 (continuous variables), or as absolute numbers and percentages (categorical variables), were compared using an unpaired Student’s t-test, Mann–Whitney U-test, or chi-square test, as appropriate. The influence of clinical factors on the risk of thyroid cancer was investigated using binary logistic regression analysis. The differences in the cancer rates between groups with determined TSH levels, considering ROC curve cutoff value, were calculated using odds ratios and 95 percent confidence intervals. Statistical analysis of the results was performed with SPSS software (Statistical Package for Social Sciences) version 18.0.

## Results

### Patient characteristics

**Fig 1:**
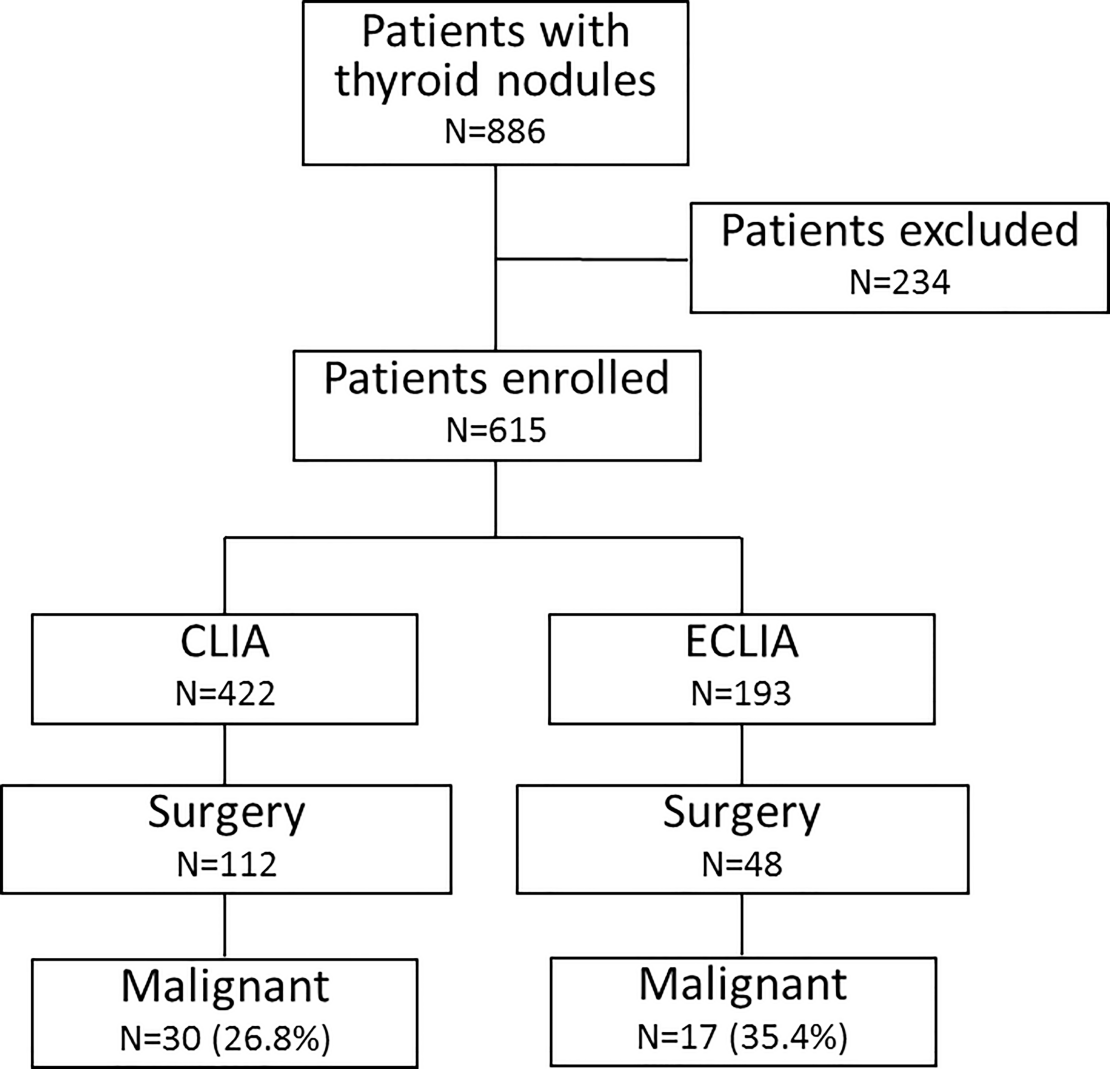
Flow chart of patients who met inclusion/exclusion criteria for the study population. CLIA, Chemiluminescent immunoassay; ECLIA, electrochemiluminscent immunoassay.

A total of 886 patients with thyroid nodules were consecutively evaluated. Of them, 234 were excluded due to thyroid hormone replacement therapy and/or a previous diagnosis of thyroid cancer, and 37 patients because they did not have available TSH measurement. Thus, 615 patients participated in the study. The mean of age of the participants was 55.9 ±14.7 years, and 544 (88.5%) were female. Solitary nodules were noted in 226 patients (36.7%). As mentioned in the Materials and Methods section, two different assays were used to measure the TSH levels during the study period. Since the reference ranges were slightly different between these assays, we have analyzed the patients separately (Fig 1). The median TSH in 422 patients evaluated by CLIA was 1.48 μU/mL (P25: 0.93 and P75: 2.31) while the median TSH was 1.55 μU/mL in 193 patients analyzed by ECLIA, (P25: 0.95 and P75: 2.80). There was no significant differences in median TSH values (P = 0.32). Indeed, the correlation between the two different TSH assays showed a strong correlation (r = 0.99). Forty-three out of 261 (16.5%) patients analyzed presented positivity to TPO-Ab.

All patients underwent US-FNBA and had their sample analyzed by cytologic and cell block diagnostic testing. The combined cytological and cell block (cyto-cell) interpretations were as follows: 5.7% (n = 35) unsatisfactory, 70.6% (n = 434) benign, 9.1% (n = 56) indeterminate, 8.1% (n = 50) follicular lesion and 6.5% (n = 40) malignant (Table 1). Surgery was indicated for 170 patients with the following cyto-cell results: 7 nondiagnostic cyto-cell blocks, 30 indeterminate lesions, 43 follicular lesions, 36 malignant lesions and 54 benign lesions, based on clinical (compressive symptoms, recent growing) and ultrasonographic characteristics (large nodules, microcalcification, irregular margin). Final histological data were available for 160 patients, 113 of whom had benign lesions (70.6%), and 47 of whom had malignant lesions (29.3.% of surgical cases and 7.6% of the sample). Of the subgroup evaluated by CLIA, 112 patients underwent surgery and 30 (26.8%) had malignant nodules while 48 out of 193 patients analyzed by ECLIA were thyroidectomized and 17 (35.4%) had thyroid cancer. The characteristics of all enrolled patients are shown in Table 1. The distribution of age, gender, and other clinical features were similar between both TSH assay groups. However, the presence of microcalcification were higher in CLIA group (19 vs. 13.5%; P = 0.00) and the distribution of cytological evaluation showed a higher prevalence of indeterminate category in ECLIA group (6.6 vs. 14.5%; P<0.001).

**Table 1:** Clinical and oncology features of patients included in the study.

	All Samples	CLIA	ECLIA	P value
	(N = 615)	(N = 422)	(N = 193)	
**Age (yr.)**	55.9 ± 14.7	56.4 ± 14.0	54.9 ± 16.0	0.25
**Female gender (%)**	544 (88.5)	378 (89.6)	166 (86)	0.20
**Family history of thyroid cancer (%)**	37/602 (6.1)	29/409 (7.1)	8/193 (4.1)	0.16
**Personal history of other neoplasia (%)**	68/604 (11.3)	48/411 (11.7)	20/193 (10.4)	0.63
**Exposure to radiation (%)**	28/601 (4.7)	23/408 (5.6)	5/188 (2.7)	0.98
**Serum TSH (μU/mL)**		1.48 (0.93–2.31)	1.55 (0.95–2.80)	0.32
**Solitary nodule (%)**	226 (36.7)	155 (36.7)	71 (36.8)	0.99
**Microcalcifications (%)**	106/483 (21.9)	80/292 (19)	26(13.5)	0.00
**FNA cyto-cell block (%)**				0.00
**I—Nondiagnostic**	35 (5.7)	20 (4.7)	15 (7.8)	
**II—Benign**	434 (70.6)	315 (74.6)	119 (61.7)	
**III—Indeterminate**	56 (9.1)	28 (6.6)	28 (14.5)	
**IV—Follicular lesion**	50 (8.1)	30 (7.1)	20 (10.4)	
**V/VI—Malignant**	40 (6.5)	29 (6.9)	11 (5.7)	

Values are the mean ± SD or median (P25-P75). CLIA, chemiluminescent immunoassay; ECLIA, electrochemiluminscent immunoassay. The TSH reference range is 0.35–5.50μU/mL by CLIA and 0.27–4.20μUI/mL by ECLIA. Solitary nodules and microcalcifications were evaluated by ultrasonography.

### TSH and thyroid cancer

In the group evaluated by CLIA, 91.7% of patients showed serum TSH levels within the normal range; 23 patients (5.5%) presented TSH levels less than 0.35 μU/mL, and 12 (2.8%) had TSH levels greater than 5.5 μU/mL. In patients analyzed by ECLIA, TSH was within the normal range in 87%; 4 (2.1%) patients showed TSH levels less than 0.27 μU/mL and 21 patients (10.9%) have TSH levels greater than 4.20 μU/mL.

**Fig 2:**
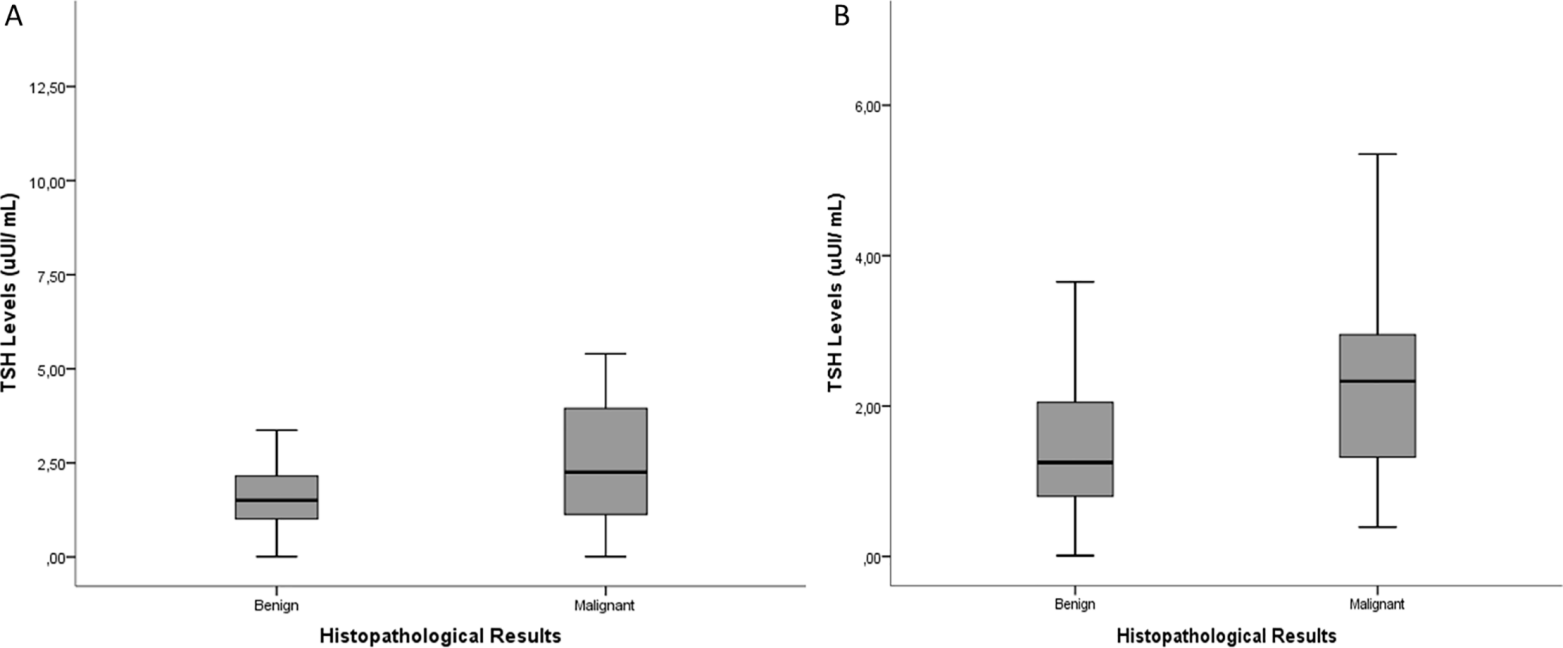
TSH level in malignant and benign thyroid nodules. CLIA, Chemiluminescent immunoassay; ECLIA, electrochemiluminscent immunoassay. Box plot illustrating the median TSH level and the TSH level quartiles and ranges in malignant and benign thyroid nodules for CLIA(A) and ECLIA (B). Patients with thyroid cancer exhibited higher median TSH levels than patients with benign nodules for both methodology. P = 0.04 (A) and P = 0.03 (B).

Noteworthy, patients with thyroid cancer exhibited higher median TSH levels than patients with benign nodules irrespectively of the assay methodology: 2.25 vs. 1.50 (CLIA;P = 0.04) and 2.33 vs. 1.27 (ECLIA; P = 0.03) (**Fig 2**;Table 2). Moreover, a family history of thyroid cancer, solitary nodules and the presence of microcalcifications were associated with malignancy (Table 2). Further analysis using binary logistic regression identified elevated TSH levels, a family history of thyroid cancer, the presence of microcalcifications and solitary nodule on ultrasonography as independent risk factors for malignancy in patients with thyroid nodules. There were no differences in age, gender, non-thyroid neoplasia history, presence of TPO-Ab positivity or previous radiation exposure history between patients with benign nodules and those with thyroid cancer.

**Table 2:** Characteristics of patients with benign and malignant nodules.

	Malignant nodule	Benign nodule	P value
	(N = 47)	(N = 113)	
**Age (yr.)**	47.9 ± 13.6	50.9 ± 14.8	0.24
**Male gender (%)**	10 (21.3)	12 (10.6)	0.07
**Family history of thyroid cancer (%)**	7 (14.9)	5 (4.4)	0.03*
**Personal history of other neoplasia (%)**	4 (8.5)	8 (7.1)	0.79
**Exposure to radiation (%)**	2 (4.2)	4 (3.5)	0.84
**Serum TSH (μU/mL)**			
**CLIA**	2.25 (1.12–4.07)	1.50 (0.99–2.17)	0.04
**ECLIA**	2.33 (1.30–3.06)	1.25 (0.79–2.10)	0.03
**Microcalcifications (%)**	21 (44.7)	16 (14.2)	0.00*
**Solitary nodule (%)**	26 (55.3)	42 (37.2)	0.03*
**TPO-Ab positively (%)**	4/22 (18.2)	6/55 (10.9)	0.39

Values are the mean ± sd or median (P25-P75). CLIA, chemiluminescent immunoassay; ECLIA, electrochemiluminscent immunoassay.The TSH reference range is 0.35–5.50μU/mL by chemiluminescent and 0.27–4.20μU/mL by electrochemiluminscent. Solitary nodules and microcalcifications were evaluated by ultrasonography (during US-FNA).

*Independent variable by binary logistic regression.

**Fig 3:**
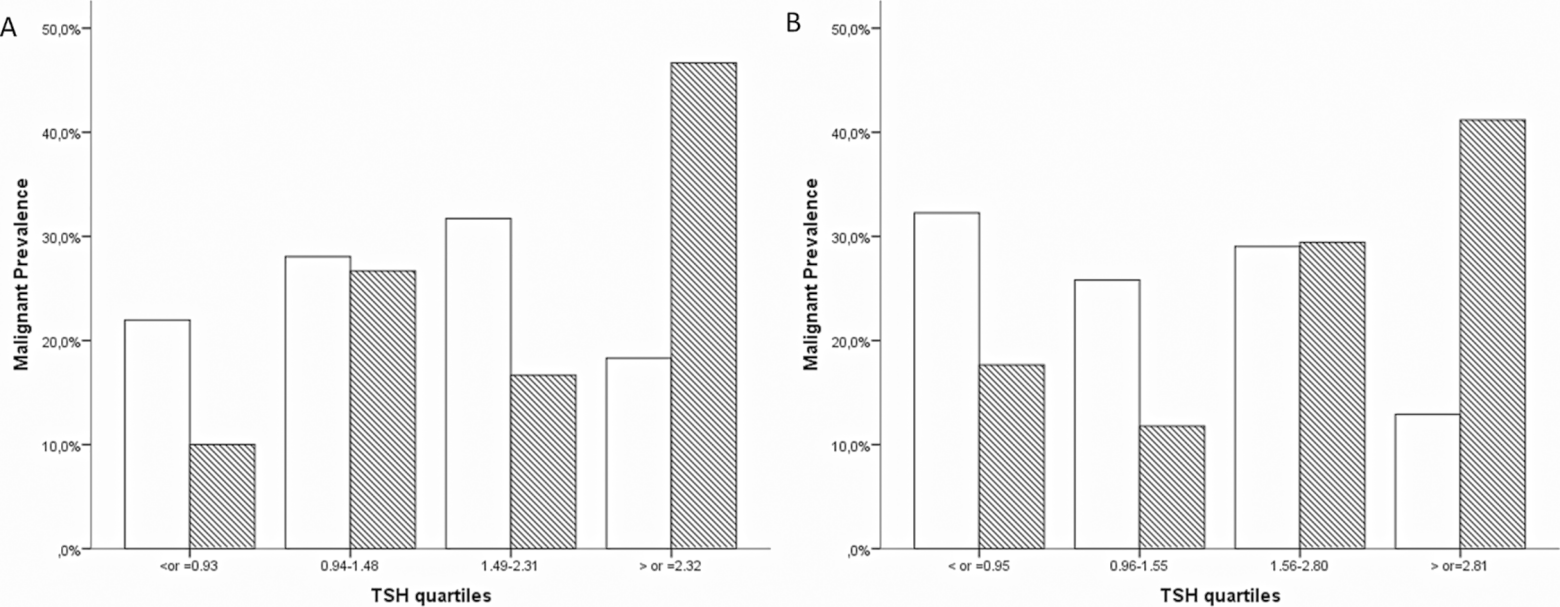
Frequencies of malignant and benign nodules according to TSH quartile. CLIA, Chemiluminescent immunoassay; ECLIA, electrochemiluminscent immunoassay. Frequencies of malignant (dashed bars) and benign (white bars) nodules according to TSH quartile for CLIA(A) and ECLIA (B). A significant increase in the prevalence of malignancy was noted in higher TSH quartiles. P<0.01.

To further explore the potential role of TSH levels as a predictor of thyroid cancer, we subdivided the sample into quartiles in accordance with the TSH level distribution of the study (≤0.93, 0.94–1.48, 1.49–2.31 and ≥2.32 μU/mL for CLIA and ≤0.95, 0.96–1.55, 1.56–2.80 and ≥2.81 μU/mL ECLIA). Interestingly, we observed that the frequencies of malignancy in each TSH quartile varies in accordance with TSH levels, for both assays (P = 0.02 and P = 0.04; respectively, Fig 3). For CLIA group, the prevalence of malignancy in patients with TSH levels in the first quartile (≤0.93 μU/mL) was 14.3%, while those in the upper quartile (≥2.32 μU/mL) exhibited a cancer prevalence of 48.3% (OR 5.6; CI 1.35–23.2)(P = 0,01). The prevalence of malignancy in patients evaluated by ECLIA with TSH levels in the first quartile (≤0.95 μU/mL) was 23%, while those in the upper quartile (≥2.81 μU/mL) exhibited a cancer prevalence of 64% (OR 5.83; CI 1.00–34.6)(P = 0,04). Of note, even within normal range of TSH, there was a significantly increased risk of thyroid cancer as TSH increased.

We subsequently performed ROC curve analysis to identify the best TSH cutoff for predicting malignancy. Remarkable, the TSH value of 2.26μU/mL was identified to both assays. Then, we perform additional analyses using TSH levels as a categorical variable, the prevalence of malignancy was higher in those patients with TSH levels >2.26 μU/mL than in patients with lower TSH levels (<2.26 μU/mL) (OR 3.87; CI 1.87–8.01)(P = 0.00). Similar results were obtained when only patients with normal TSH levels were analyzed (OR 3.42 CI 1.62–7.24) (P = 0.00).

Among the 47 patients with thyroid cancer, three cases harbor medullary thyroid carcinoma and 44 differentiated thyroid carcinoma (DTC). Among patients with DTC, 11 cases were microcarcinoma (25.0%). There were no differences in prevalence of TSH >2.26 μU/mL or distribution of TSH quartile between patients with or without microcarcinoma.

## Discussion

In the present prospective study, we demonstrated that patients with malignant thyroid nodules presented higher serum TSH levels than patients with benign nodules. Accordingly, the prevalence of malignancy was higher in subjects with TSH levels >2.26 μU/mL, as defined via ROC curve analyses. Remarkably, additional analyses using TSH levels as a categorical variable show that the risk of malignancy was higher in patients with TSH levels in upper quartile as compared with patients with lower TSH levels in two different methodologies.

TSH is a major thyroid cell growth factor, while TSH signaling pathway activation may be required for the expression of other growth factors, receptors, and proto-oncogenes[28–30]. Accordingly, TSH suppression is an important therapeutic tool of clinical thyroid cancer management[5,7]. In the last years, several studies have addressed the role of TSH as a predictor of thyroid nodule malignancy but the results are still open to discussion. Here, we have demonstrated that patients with higher TSH levels have increased risk for malignancy. Remarkable, as TSH quartiles increased, the likelihood of malignancy rose, and the odds ratio of thyroid cancer in patients with TSH levels in the upper quartile was 5-fold higher than in those patients with TSH levels in the first quartile (Fig 3). Similar results were observed using the ROC analyses to define the TSH levels. Our observations are in agreement with previous studies [21–25]although contrast with the data from the EPIC study[26]. Noteworthy, however, the EPIC was a case-control study that includes only cancer-free subjects which might be a limiting when compared with our study.

Despite the consistent association between higher TSH levels and malignant nodules shown in most series, including this one, an optimal TSH cutoff value for predicting the risk of cancer has not been yet identified. Indeed, the lack of previous studies validating nomograms or equations intended to determine an optimal TSH cutoff value has limited the use of serum TSH levels as a malignancy predictor. Here, we found the TSH cutoff value (≥2.26 μU/mL) using the best point of a ROC curve for the two different TSH assays used during the study period. Some authors have suggested that while no consensus exists regarding a TSH cutoff value, it may be practical to preferentially perform US-FNAB in nodules less than 1.5 or 2.0 cm exhibiting patterns arousing low or very low suspicion for malignancy on ultrasonography[2,25]. Haymart et al. suggested TSH may play a key role in optimizing surgical interventions when aspirates are suspicious for malignancy[23]. These recommendations might support an adjunctive role for TSH levels when evaluating thyroid nodules.

This study has several strengths, as it included a large number of patients with thyroid nodules who were evaluated at a single institution and did not exclude patients with abnormal TSH levels, which enhances the external validity of its findings and increases the clinical applicability of its data. Also, two TSH methodologies were analyzed with consistent results. Thus, the results presented here may have important clinical implications, since its indicate that TSH levels may help on the diagnosis strategy, in conjunction with clinical, ultrasonographic and cytological features. However, as noted above, TSH should not be used for diagnostic decision-making in isolation. Also, although we demonstrated a prospective study data, the design of our study was not delineated for evaluate the TSH as a causative role in thyroid cancer pathogenesis. In this view, we do not recommend screening for thyroid cancer in patients with chronic TSH elevations nor suppressive treatment for subclinical hypothyroidism and for benign nodular disease.

## Conclusions

Higher serum TSH levels are associated with an increased risk of thyroid cancer in patients with thyroid nodules. The use of TSH as an adjunctive diagnostic test for stratifying the risk of malignancy associated with thyroid nodules may have value to decision-making on diagnostic approaches.
